# Fabrication of SrGe_2_ thin films on Ge (100), (110), and (111) substrates

**DOI:** 10.1186/s11671-018-2437-1

**Published:** 2018-01-16

**Authors:** T. Imajo, K. Toko, R. Takabe, N. Saitoh, N. Yoshizawa, T. Suemasu

**Affiliations:** 1 Institute of Applied Physics, University of Tsukuba, 1-1-1 Tennodai, Tsukuba, Ibaraki 305-8573 Japan; 2 Electron Microscope Facility, TIA, AIST, 16-1 Onogawa, Tsukuba, 305-8569 Japan

**Keywords:** Germanides, Epitaxy, Nanostructures, Solar cells

## Abstract

Semiconductor strontium digermanide (SrGe_2_) has a large absorption coefficient in the near-infrared light region and is expected to be useful for multijunction solar cells. This study firstly demonstrates the formation of SrGe_2_ thin films via a reactive deposition epitaxy on Ge substrates. The growth morphology of SrGe_2_ dramatically changed depending on the growth temperature (300−700 °C) and the crystal orientation of the Ge substrate. We succeeded in obtaining single-oriented SrGe_2_ using a Ge (110) substrate at 500 °C. Development on Si or glass substrates will lead to the application of SrGe_2_ to high-efficiency thin-film solar cells.

## Background

Alkaline-earth silicides have been widely investigated because of their useful functions for many technological applications such as solar cells [1–3], thermoelectrics [4–6], and optoelectronics [7–9]. However, the study of germanides has not been active compared to that of silicides even though some studies have predicted interesting electrical and optical properties for germanides [10–16].

SrGe_2_ is one of the alkaline-earth germanides. Theoretical and experimental studies of bulk SrGe_2_ have revealed the following properties [12–16]: (i) a BaSi_2_-type structure (orthorhombic, space group: ${D}_{2h}^{16}- Pnma$, no. 62, *Z* = 8), (ii) an indirect transition semiconductor with a band gap of approximately 0.82 eV, and (iii) an absorption coefficient of 7.8 × 10^5^ cm^−1^ at 1.5 eV photon, which is higher than that of Ge (4.5 × 10^5^ cm^−1^ at 1.5 eV photon). These properties mean that SrGe_2_ is an ideal material for use in the bottom cell of high-efficiency tandem solar cells. Therefore, the fabrication of a SrGe_2_ thin film on arbitrary substrates would allow thin-film tandem solar cells simultaneously achieving high conversion efficiency and low process cost.

We fabricated thin-film BaSi_2_, having the same structure as SrGe_2_, on Si (111) and Si (001) substrates using a two-step method: a BaSi_2_ template layer was formed via reactive deposition epitaxy (RDE), which is a Ba deposition with heated Si substrates, followed by molecular beam epitaxy (MBE) [17, 18]. This resulted in high-quality (100)-oriented BaSi_2_ thin films with a long minority carrier life time [19, 20], leading to a large minority carrier diffusion length [21] and a high photoresponsivity at 1.55 eV [22]. The heterojunction solar cell with the p-BaSi_2_/n-Si structure allowed for a conversion efficiency of 9.9%, the highest value ever reported for semiconducting silicides [23]. These impressive results on the BaSi_2_ thin films and the attractive properties of bulk SrGe_2_ strongly motivated us to fabricate SrGe_2_ thin films.

The two-step method consisting of RDE and MBE to form BaSi_2_ thin films on Si substrates is applicable to fabricating SrGe_2_ thin films on Ge substrates because these materials have the same crystal structure [14]. In this study, we tried to form SrGe_2_ on Ge (100), (110), and (111) substrates using RDE to explore the possibility of SrGe_2_ thin-film formation.

## Experimental

A molecular beam epitaxy system (base pressure, 5 × 10^−7^ Pa) equipped with a standard Knudsen cell for Sr and an electron-beam evaporation source for Si were used in this investigation. Sr was deposited on Ge (100), (110), and (111) substrates where the substrate temperature (*T*_sub_) ranged from 300 to 700 °C. Before the deposition, the Ge substrate was cleaned using a 1.5% HF solution for 2 min and a 7% HCl solution for 5 min. The deposition rate and time of Sr were, respectively, 0.7 nm/min and 120 min for Ge (001), 1.4 nm/min and 30 min for Ge (011), and 1.3 nm/min and 60 min for Ge (111). The deposition rate varied depending on the amount of the Sr source because the Knudsen cell temperature was fixed at 380 °C. After that, 5-nm-thick amorphous Si was deposited at room temperature to protect the RDE layer from oxidation because Sr−Ge compounds are easily oxidized by air. The crystallinity of the sample was evaluated using reflection high-energy electron diffraction (RHEED) and X-ray diffraction (XRD; Rigaku Smart Lab) with Cu Kα radiation. In addition, the surface morphology was observed using scanning electron microscopy (SEM; Hitachi SU-8020) and transmission electron microscopy (TEM; FEI Tecnai Osiris) operated at 200 kV, equipped with an energy-dispersive X-ray spectrometer (EDX), and a high-angle annular dark-field scanning transmission electron microscopy (HAADF-STEM) system with a probe diameter of ~ 1 nm.

## Results and Discussion

**Fig. 1:**
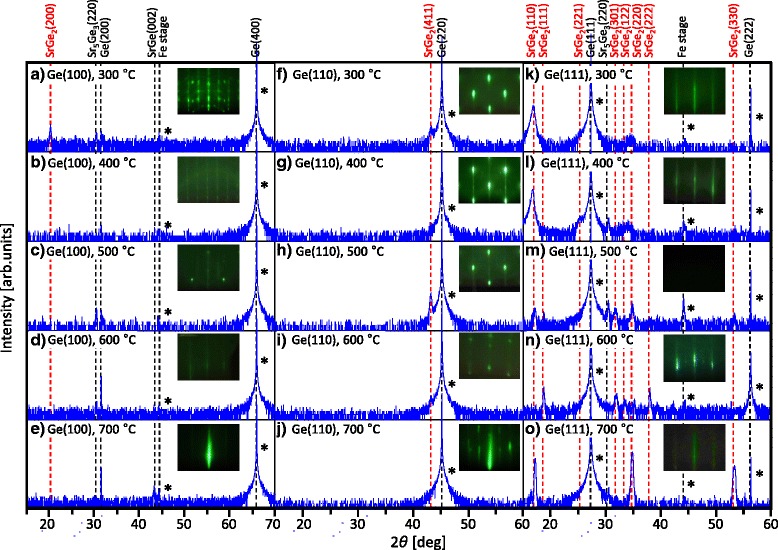
RHEED and *θ*–2*θ* XRD patterns of the samples after the Sr deposition. The crystal orientation of the Ge substrate is **a**−**e** (100), **f**−**j** (110), and **k**−**o** (111). *T*_sub_ is ranged from 300 to 700 °C for each substrate. The peaks corresponding to SrGe_2_ are highlighted in red

**Fig. 2:**
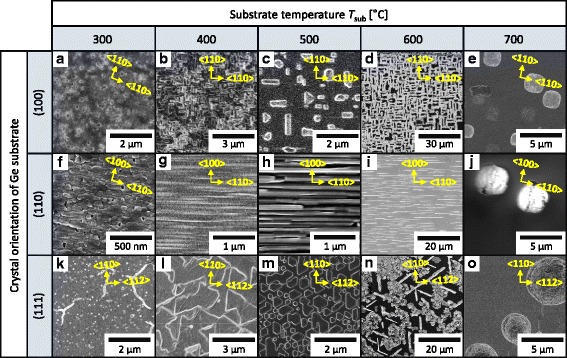
SEM images of the samples after the Sr deposition. The crystal orientation of the Ge substrate is **a**−**e** (100), **f**−**j**, (110), and **k**−**o** (111). *T*_sub_ is ranged from 300 to 700 °C for each substrate. The arrows in each image show the crystal directions of the Ge substrates

Figure 1 shows the RHEED and *θ*–2*θ* XRD patterns of the samples after the Sr deposition. For all samples, streaky or spotted RHEED patterns were observed after the Sr deposition, implying the epitaxial growth of Sr−Ge compounds. For the samples with a Ge (100) substrate, peaks from Sr_5_Ge_3_ appear for all *T*_sub_ (Fig. 1a−e). In addition, peaks from SrGe appear for *T*_sub_ = 600 and 700 °C (Fig. 1d, e). Only the sample with *T*_sub_ = 300 °C exhibits the peak from SrGe_2_ (Fig. 1a), the target material in this study. Figure 1a shows that the sample with *T*_sub_ = 300 °C contains preferentially [100]-oriented SrGe_2_ and [220]-oriented Sr_5_Ge_3_. The peak derived from the substrate, Ge (200), is more noticeable for higher *T*_sub_. This behavior is related to the surface coverage of Sr–Ge compounds on the substrate as revealed in Fig. 2. For the samples with a Ge (110) substrate, no peaks other than those from SrGe_2_ (411) and the Ge substrate are observed for *T*_sub_ = 300−600 °C (Fig. 1f−i). The peak from SrGe_2_ (411) exhibits the highest intensity for *T*_sub_ = 500 °C (Fig. 1h), suggesting that the sample with *T*_sub_ = 500 °C contains single-composition SrGe_2_ with high [411] orientation. For the samples with a Ge (111) substrate, the peaks from SrGe_2_ appear for all *T*_sub_ (Fig. 1k−o). The samples with *T*_sub_ = 300, 400, 500, and 700 °C exhibit [110]-oriented SrGe_2_ (Fig. 1k–m, o), while the SrGe_2_ peaks for *T*_sub_ = 300 and 400 °C are quite broad. The samples with *T*_sub_ = 500 and 600 °C exhibit multi-oriented SrGe_2_ (Fig. 1m, n). In addition, the small peak from Sr_5_Ge_3_ (220) appears for *T*_sub_ = 400, 500, and 700 °C (Fig. 1l, m, o). Therefore, the growth morphology of Sr–Ge compounds on a Ge substrate dramatically changes depending on the growth temperature and the crystal orientation of the substrate. This behavior is likely related to the surface energy of the Ge substrate depending on the crystal orientation [24] and the balance of the supply rate of Ge atoms from the substrate and the evaporation rates of Sr atoms from the sample surface.

Figure 2 shows SEM images of the sample surfaces. It is seen that the substrates are mostly covered by Sr−Ge compounds for *T*_sub_ = 300 °C (Fig. 2a, f,k). For *T*_sub_ = 400, 500, and 600 °C, we can observe the unique patterns reflecting the crystal orientation of the substrates, that is, twofold symmetry for Ge (100) (Fig. 2b−d), onefold symmetry for Ge (110) (Fig. 2g−i), and threefold symmetry for Ge (111) (Fig. 2l−n). These patterns can also be seen for silicides on Si substrates [1, 25] and ensure the epitaxial growth of Sr−Ge compounds on the Ge substrates. The samples with *T*_sub_ = 700 °C exhibit dot patterns, suggesting that the Sr atoms migrated rapidly and/or evaporated due to the high *T*_sub_. These SEM results account for the streaky or spotted RHEED patterns in Fig. 1. Therefore, we succeeded in obtaining single-oriented SrGe_2_ using a Ge (110) substrate with *T*_sub_ = 500 °C, while for Ge (100) and Ge (111) substrates, multiple-oriented SrGe_2_ or other Sr–Ge compounds were obtained.

**Fig. 3:**
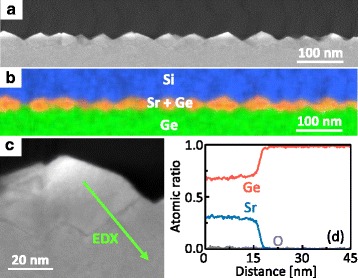
HAADF-STEM and EDX characterization of the SrGe_2_ thin film grown on the Ge (110) substrate at 500 °C. **a** HAADF-STEM image. **b** EDX elemental map from the region shown in panel **a**. **c** Magnified HAADF-STEM image. **d** Elemental composition profile obtained by a STEM-EDX line scan measurement along the arrow in panel (**c**)

We evaluated the detailed cross-sectional structure of the sample with a Ge (110) substrate and *T*_sub_ = 500 °C. To prevent oxidation of the SrGe_2_, a 100-nm-thick amorphous Si layer was deposited on the sample surface. The HAADF-STEM image in Fig. 3a and the EDX mapping in Fig. 3b show that the Sr–Ge compound is formed on nearly the entire surface of the Ge substrate. The magnified HAADF-STEM image in Fig. 3c shows that the Sr–Ge compound digs into the Ge substrate, which is a typical feature of RDE growth [17, 18]. The elemental composition profile in Fig. 3d shows that Sr and Ge exist with a composition of 1:2. The results in Figs. 1 and 3 confirm the formation of SrGe_2_ crystals.

**Fig. 4:**
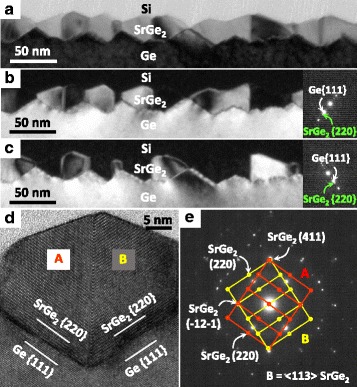
TEM characterization of the SrGe_2_ thin film grown on the Ge (110) substrate at 500 °C. **a** Bright-field TEM image. **b**, **c** Dark-field TEM images using the SrGe_2_ {220} plane reflection shown in each diffraction pattern. **d** High-resolution lattice image showing SrGe_2_ crystals. **e** SAED pattern showing the SrGe_2_ 〈113〉 zone axis, taken from the region including SrGe_2_ crystals and the Ge substrate

The bright-field TEM image in Fig. 4a and the dark-field TEM images in Fig. 4b, c show that while SrGe_2_ is epitaxially grown on the Ge substrate, it has two orientations in the in-plane direction. The lattice image in Fig. 4d clearly shows two SrGe_2_ crystals (A and B) and a grain boundary between them. The selected area diffraction pattern (SAED) in Fig. 4e shows diffraction patterns corresponding to two SrGe_2_ crystals (A and B). Figure 4d, e also shows that the Ge (111) plane and the SrGe_2_ (220) plane are parallel in each crystal. These results suggest that the SrGe_2_ crystals A and B epitaxially grew from the Ge (111) plane of the substrate and then collided with each other. No defects, such as dislocations or stacking faults, were found in the SrGe_2_ besides the grain boundary. Therefore, high-quality SrGe_2_ crystals were successfully obtained via RDE growth on a Ge(110) substrate.

## Conclusions

We successfully formed thin films of SrGe_2_ via RDE growth on Ge substrates. The growth morphology of SrGe_2_ dramatically changed depending on the growth temperature and the crystal orientation of the Ge substrate. Even though multiple-oriented SrGe_2_ or other Sr–Ge compounds were obtained for Ge (100) and Ge (111) substrates, we succeeded in obtaining single-oriented SrGe_2_ by using a Ge (110) substrate at a growth temperature of 500 °C. Transmission electron microscopy revealed that the SrGe_2_ thin film on the Ge (110) substrate had no dislocation at the substrate interface. Therefore, we demonstrated that high-quality SrGe_2_ thin films can be produced. At present, we are investigating the characterization of the SrGe_2_ thin films and their development on Si and glass substrates for the application of SrGe_2_ to near infrared light absorption layers of multijunction solar cells.

## Abbreviations

EDX: Energy-dispersive X-ray spectrometer
HAADF-STEM: High-angle annular dark-field scanning transmission electron microscopy
MBE: Molecular beam epitaxy
RDE: Reactive deposition epitaxy
RHEED: Reflection high-energy electron diffraction
SEM: Scanning electron microscopy
TEM: Transmission electron microscopy
*T*_sub_: Substrate temperature
XRD: X-ray diffraction
